# Changes in Prevalence and Health Checkup Coverage Rate of Chronic Kidney Disease (CKD) after Introduction of Prefecture-Wide CKD Initiative: Results of the Kagawa Association of CKD Initiatives

**DOI:** 10.3390/jpm11111121

**Published:** 2021-10-30

**Authors:** Tadashi Sofue, Taiga Hara, Yoko Nishijima, Satoshi Nishioka, Hiroyuki Watatani, Masahito Yamanaka, Norihiro Takahashi, Akira Nishiyama, Tetsuo Minamino, Kagawa Association of Chronic Kidney Disease Initiatives

**Affiliations:** 1 Department of Cardiorenal and Cerebrovascular Medicine, Kagawa University, Takamatsu 761-0793, Kagawa, Japan; minamino.tetsuo.gk@kagawa-u.ac.jp; 2 Department of Medicine for Community Healthcare Revitalization, Kagawa University, Takamatsu 761-0793, Kagawa, Japan; hara.taiga@kagawa-u.ac.jp; 3 Department of CardioRenal Disease Regional Medicine, Kagawa University, Takamatsu 761-0793, Kagawa, Japan; nishijima.yoko@kagawa-u.ac.jp; 4 Department of Nephrology, Takamatsu Red Cross Hospital, Takamatsu 760-0017, Kagawa, Japan; nsatoshi2400@yahoo.co.jp; 5 Department of Nephrology and Rheumatology, Kagawa Prefectural Central Hospital, Takamatsu 760-8557, Kagawa, Japan; watatani@pa3.so-net.ne.jp; 6 Department of Urology, Takamatsu Red Cross Hospital, Takamatsu 760-0017, Kagawa, Japan; ymasahito21@gmail.com; 7 Department of Internal medicine, Shido-Akiyama Clinic, Takamatsu 769-2101, Kagawa, Japan; ntaka3658@yahoo.co.jp; 8 Department of Pharmacology, Kagawa University, Takamatsu 761-0793, Kagawa, Japan; nishiyama.akira@kagawa-u.ac.jp

**Keywords:** chronic kidney disease, health checkup, medical costs, coverage rate

## Abstract

The National Health Insurance (NHI) special health checkup system in Japan targets the NHI population aged 40–74 years. Since 2015, the Kagawa NHI special health checkup was initiated in a prefecture-wide chronic kidney disease (CKD) initiative, including renal examination as an essential item in NHI health checkups. Here, we aimed to investigate the effects of the prefecture-wide CKD initiative. We conducted a retrospective cohort survey using the Kagawa National Health Insurance database created by the Kagawa National Health Insurance Organization. Results of the NHI health checkup (2015–2019) and prefecture-wide outcomes (2013–2019) were analyzed. The prevalence of CKD among examinees who underwent the NHI health checkup increased from 17.7% in 2015 to 23.2% in 2019. The percentage of examinees who completed a medical visit was 29.4% in 2015. After initiation of the initiative, the NHI health checkup coverage rate increased significantly, from a mean (standard deviation) of 40.8% (0.4%) to 43.2% (1.1%) (*p* = 0.04). After the start of the CKD initiative, we found an increase in the prevalence of CKD and the NHI health checkup coverage rate.

## 1. Introduction

Chronic kidney disease (CKD) may develop into end-stage renal disease (ESRD), which requires dialysis or kidney transplantation. CKD is a serious public health problem, with an enormous economic burden [1,2]. It is estimated that 10 to 12% of Japanese adults (over 10 million people) have CKD [3,4,5]. One beneficial approach to reducing the risk of incident ESRD is screening for CKD [6]. In 2008, the Japanese Ministry of Health, Labor, and Welfare (MHLW) introduced a nationwide screening program to identify individuals aged 40–74 years with high levels of obesity and cardiovascular risks (known as metabolic syndrome), with the aim of providing health guidance to reduce weight and improve cardiovascular risk [7,8]. However, up to 2019, kidney-specific testing (kidney function and proteinuria) had not been performed on all examinees receiving health checkups. A previous Japanese study suggested that CKD screening using dipstick urinalysis and/or serum creatinine (Cr) measurement was a cost-effective approach to preventing progression to ESRD [9]. In response to this, in 2019, the MHLW encouraged adults aged ≥40 years to undergo CKD screening through the health checkup program, which includes mandatory dipstick urinalysis and optional serum Cr measurement [10].

The Kagawa Special Health Checkup System targets the National Health Insurance (NHI) population aged 40–74 years. Nephrologists in Kagawa Prefecture established the Kagawa Association of Chronic Kidney Disease Initiatives in 2012 and initiated a prefecture-wide CKD initiative, which includes both glomerular filtration rates (eGFR) and urinary examination as essential parts of the NHI health checkup. As part of this initiative, recommendations are given to patients for attending lifestyle guidance classes for CKD or visiting a general physician [11]. 

In this population-based study, we aimed to investigate the effects of the prefecture-wide CKD initiative on the prevalence and medical costs of CKD and the NHI health checkup coverage rate.

## 2. Materials and Methods

### 2.1. Overview of Prefecture-Wide CKD Initiative

The NHI mainly covers self-employed individuals, retirees, and their non-working dependents aged <75 years, and the Medical Care System for the Elderly covers adults aged >75 years or aged 65–74 years who have certain disabilities. Since 2015, CKD staging using CKD classification has been conducted for examinees who undergo NHI health checkups in Kagawa Prefecture, using dipstick proteinuria and serum Cr sampling.

Suspected CKD is defined as eGFR <60 mL/min/1.73 m^2^ or results of qualitative analysis of urinary protein (UP) ≥1+ in a health checkup. Moderate-to-severe CKD is defined as eGFR <50 mL/min/1.73 m^2^ (40 mL/min/1.73 m^2^ at age ≥ 70 years) and/or ≥UP 2+. Early CKD is defined as eGFR of 50 to <60 mL/min/1.73 m^2^ (or 40 to <60 mL/min/1.73 m^2^ at age ≥70 years and/or ≥UP 1+. As these criteria are based on the Clinical Practice Guidebook for the Diagnosis and Treatment of Chronic Kidney Disease 2012, published by the Japanese Society of Nephrology [12], they do not completely meet the criteria for CKD. 

**Figure 1:**
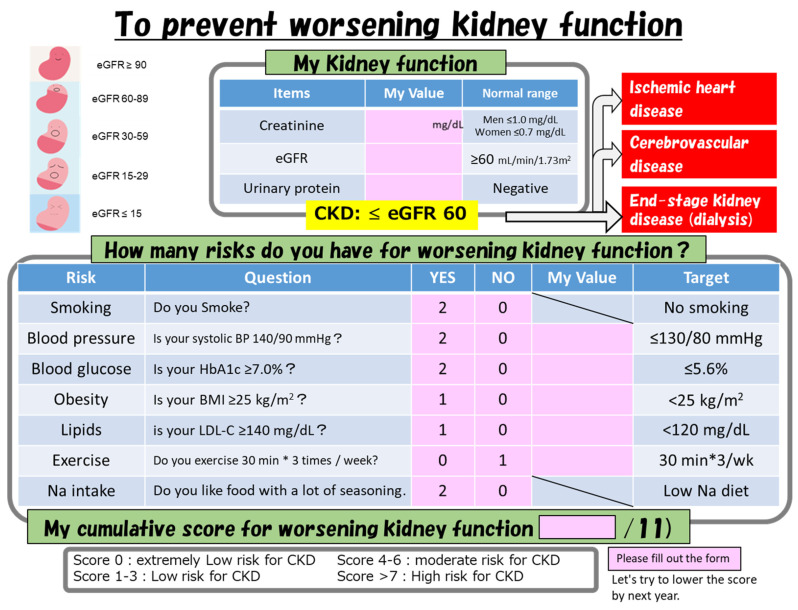
Risk factor list chart for CKD used in the participatory structured group education program: eGFR, estimated glomerular filtration rate; Na, sodium; BP, blood pressure; HbA1c, glycated hemoglobin; BMI, body mass index; LDL-C, low-density lipoprotein cholesterol; CKD, chronic kidney disease.

Examinees with early CKD are encouraged to participate in CKD-specific lifestyle guidance classes provided at each city hall for individuals with a recommendation letter from the Health and Welfare Department of each city. Lifestyle guidance for CKD is performed via a participatory structured group educational (SGE) program [11]. The SGE program includes lifestyle guidance from public health nurses, dietary guidance from nutritionists, and structured group work. In this SGE program, attendees discuss and identify their remaining risk factors using the risk factor list chart (Figure 1 and Figure S1).

Examinees with moderate-to-severe CKD are not recommended lifestyle guidance but instead are encouraged to undergo a medical visit with a general physician in their region and are given a recommendation letter. To standardize CKD management among general physicians, a lecture is held about CKD management, and general physicians are requested to provide medical examinations to patients with CKD who participate in the program. In the lecture, a medical expenses assistance program for CKD, which has been independently designated an intractable disease by Kagawa Prefecture, is introduced to general physicians. We also created a program for CKD management and a list of nephrologists in Kagawa Prefecture, and we sent these to all clinics that provided health checkups. Nearly all general physicians who managed patients with CKD followed this program and cooperated in providing medical visits and recommending consultations with nephrologists for patients with eGFR ≤ 45 mL/min/1.73 m^2^ or UP > 0.5 g/gCr at the time of the medical visit. 

We used banners and roadside promotional campaigns to inform the public about the CKD-specific checkups provided as part of NHI health checkups (Figure S2). Kagawa Prefecture is the only prefecture in Japan to designate CKD as an intractable disease, regardless of the primary disease, and to provide assistance with medical expenses [13]. The criteria for application of the intractable disease system for CKD in Kagawa Prefecture are eGFR ≤ 50 mL/min/1.73 m^2^ or serum Cr ≥ 2 mg/dL.

### 2.2. Study Design

We conducted a retrospective cohort study using the Kagawa NHI database created by the Kagawa National Health Insurance Organization. We conducted a retrospective observational study using health checkup results and receipt data extracted from the National Health Insurance (Kokuho) Database system developed by the All-Japan Federation of National Health Insurance Organizations. The prevalence of CKD, the CKD lifestyle guidance attendance rate, and the rate of clinic visits after initiation of the prefecture-wide CKD initiative were analyzed using the results from 2015 to 2019. The prevalence of CKD was defined as the total number of patients with early CKD and moderate-to-severe CKD. The attendance rate for CKD lifestyle guidance was calculated as the number of examinees who completed CKD lifestyle guidance classes divided by the number with early CKD. The medical visit rate was calculated as the number of examinees who returned their recommendation letter signed by an attending physician, indicating a completed medical visit, divided by the number with moderate-to-severe CKD. The rate of consultation with a nephrologist was determined using the number of recommendation letters returned with the box “nephrologist consultation” checked. The NHI health checkup coverage rate was calculated as the number of examinees undergoing an NHI health checkup divided by the target population for NHI health checkups each year. The NHI health checkup coverage rate was analyzed from 2013 to 2019. 

Data obtained in the NHI health checkup were eGFR and UP. Kidney function was determined using eGFR, which was calculated with the Modification of Diet in Renal Disease Study equation, modified for Japanese individuals [14]. UP was evaluated with dipstick urinalysis.

All procedures performed in this study and informed consent forms were reviewed and approved by the Ethics Committee of Kagawa University (#2021-111) and were consistent with the 1964 Declaration of Helsinki and its later amendments or comparable ethical standards.

### 2.3. Data Source

The proportion of total NHI medical costs for renal disease, the total number of patients receiving dialysis, the number of patients newly receiving dialysis per year, the average age of patients newly receiving dialysis in Kagawa Prefecture, and the number of recipients of the intractable disease system for CKD in Kagawa Prefecture were analyzed from 2013 to 2019. Medical treatment rates for hypertension and diabetes were analyzed from 2017 to 2020. We assessed the degree of change in these factors by comparing the mean values of these factors prior to (2013–2014) and after (2015–2019) the start of the prefecture-wide CKD initiative.

NHI medical costs and medical treatment rates for hypertension and diabetes were calculated using a Kokuho total System (INTEC Inc., Toyama, Japan). Disease statistics were created by matching the receipt data with International Classification of Disease Tenth Revision (ICD-10) codes and assigning these to the disease classification for the social insurance chapter. NHI medical costs related to renal disease were extracted using the following codes [15]: 1401, glomerular disease, tubulointerstitial disease; 1402, kidney failure. The proportion of total NHI medical costs related to renal disease was defined as medical costs related to the renal disease divided by total NHI medical costs. Medical treatment rates for hypertension and diabetes (%) were calculated using the percentage of NHI-insured individuals who received treatment for hypertension or diabetes (n/number of insured). The presence of hypertension and diabetes was determined using Diagnosis Procedure Combination codes.

The number of recipients of the intractable disease system for CKD was obtained from Kagawa Prefecture. Data for the total number of patients receiving dialysis in Kagawa Prefecture, the number of patients newly receiving dialysis per year, and the average age of patients newly receiving dialysis were provided by the Japanese Society for Dialysis Therapy. The interpretation and reporting of these data are the responsibility of the authors and should in no way be seen as an official policy or interpretation of the Japanese Society for Dialysis Therapy. These prefecture-wide data include data from NHI and late-stage medical care insurance as well as data from employee insurance.

### 2.4. Statistical Analysis

All statistical analyses were performed using IBM SPSS software version 23.0 for Windows (IBM Corp., Armonk, NY, USA), and *p* < 0.05 was considered to indicate statistical significance. Values are presented as mean with standard deviation (SD) or number with percentage, as appropriate. Variables were compared between groups using the χ^2^ test for categorical variables, as well as Student’s *t*-test, one-way analysis of variance, or Mann–Whitney *U* test for continuous variables.

## 3. Results

### 3.1. Changes in the Prevalence of CKD

**Figure 2:**
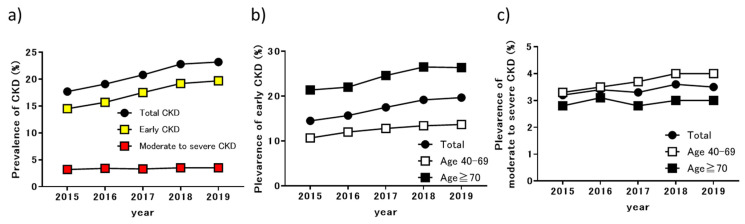
Changes in the prevalence of CKD among examinees undergoing NHI health checkups: (**a**) changes in CKD prevalence each year; (**b**) changes in the prevalence of early CKD stratified by age; (**c**) changes in the prevalence of moderate-to-severe CKD stratified by age. CKD, chronic kidney disease; NHI, national health insurance.

The changes in the prevalence of CKD among examinees who received NHI health checkups after the start of the CKD initiative in 2015 are shown in Figure 2. In 2015, 17.7% of examines were identified as having CKD, 14.5% with early CKD, and 3.2% with moderate-to-severe CKD. The prevalence of CKD among examinees in the NHI health checkups increased each year, reaching 23.2% (19.7% for early CKD and 3.5% for moderate-to-severe CKD) in 2019 (Figure 2a). Moderate to severe CKD did not show a significant increase, but early CKD increased significantly over the years (*p* < 0.01). The prevalence of early CKD was higher in all years among examinees aged ≥70 years, in comparison with those aged 40–69 years (Figure 2b). The increase in the prevalence of early CKD was more apparent in patients aged ≥ 70 years, but the prevalence of early CKD also increased gradually in patients aged 40–69 years. The prevalence of moderate-to-severe CKD was higher in all years for examinees aged 40–69 years, compared with those aged ≥70 years. The prevalence of moderate-to-severe CKD increased yearly among examinees aged 40–69 years. Additionally, there was no apparent difference in the prevalence of CKD among 17 cities and towns in 2017 (Figure S3). The prevalence of early CKD in rural areas (Ayagawa, Utadu, and Manno Town) tended to be higher than those in urban areas (Takamatsu and Marugame City), but the prevalence of moderate-to-severe CKD was similar. There was no obvious association between the percentage of examinees aged ≥70 years and the prevalence of CKD.

### 3.2. Distribution of CKD Categories by Age

**Figure 3:**
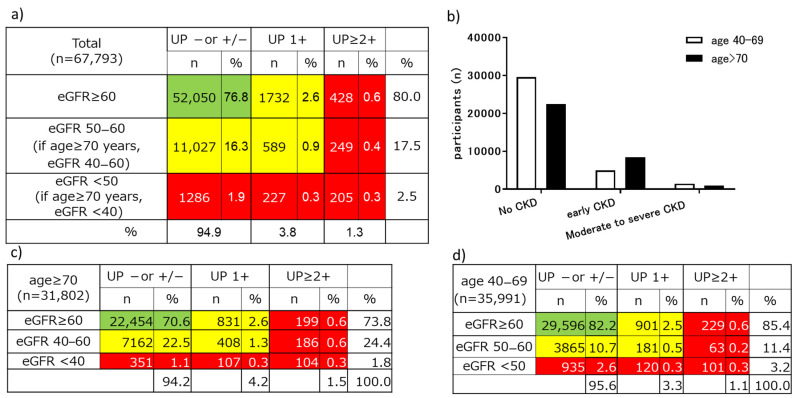
Distribution of CKD categories in the NHI-specific health examination during 2019: (**a**) distribution of CKD categories in all age groups; (**b**) distribution of CKD categories by age group; (**c**) distribution of CKD categories in the age group ≥70 years; (**d**) distribution of CKD categories in the age group 40 to 69 years. CKD, chronic kidney disease; NHI, National Health Insurance; eGFR, estimated glomerular filtration rate; UP, urinary protein.

The distribution of CKD categories by age in the NHI-specific health examination in 2019 is shown in Figure 3. The prevalence of CKD was 29.4% among examinees aged ≥70 years and 17.8% among examinees aged 40–69 years (Figure 3a). There was a significant difference in the distribution of CKD prevalence between examinees aged ≥70 years and those aged 40–69 years (*p* < 0.001, Figure 3b). In both the early and moderate-to-severe CKD groups, there were more examinees with negative proteinuria or +/− proteinuria than those with proteinuria ≥1+. This trend was similar among examinees aged ≥70 years and those aged 40–69 years (Figure 3c,d).

### 3.3. Response to the Prefecture-Wide CKD Initiative

**Figure 4:**
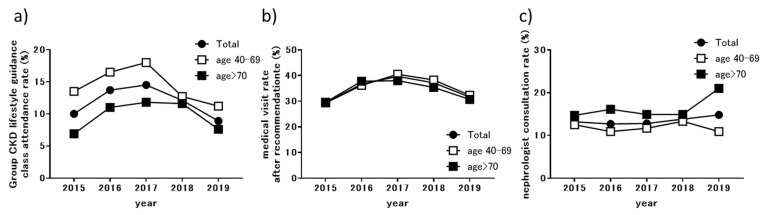
Response of examinees undergoing NHI health checkups to prefecture-wide CKD initiative: (**a**) group CKD lifestyle guidance class attendance rates; (**b**) medical visit rates after recommendation; (**c**) nephrologist consultation rates. NHI, National Health Insurance; CKD, chronic kidney disease.

The response of examinees who underwent NHI health checkups to the prefecture-wide CKD initiative is shown in Figure 4. Among examinees, those with early CKD were encouraged to participate in group lifestyle guidance classes and received a recommendation letter. In 2015 (the first year), we sent recommendation letters to 10,414 recipients, and 1043 (10.0%) attended lifestyle guidance classes organized in their city or town (Figure 4a). The rate of participation in these guidance classes increased until the third year of the program (2017) and then showed a gradual downward trend. The rate of participation in lifestyle guidance classes was higher among examinees aged 40–69 years than among those aged ≥70 years in all years. The percentage of municipalities providing CKD-specific lifestyle guidance classes was 47% in 2015, 76% in 2016, and 100% in 2017 and later, indicating that prefecture-wide lifestyle guidance classes were successfully implemented until the third year of the program.

The percentage of NHI health checkup examinees who completed a medical visit among those who were sent a medical visit recommendation letter was 29.4% (666/2266) in the first year. This rate increased gradually until the third year of the program and has remained at the initial level (29.4% in 2015) since then. The annual percentage of patients who were referred to a nephrologist after visiting a general physician, based on a recommendation letter, is shown in Figure 4c. In 2015, 666 patients with recommendation letters visited a general physician, of which 88 (56 aged 40–69 years and 32 aged ≥70 years) were referred to a nephrologist. The nephrologist referral rate did not vary from the start of the initiative until 2019 when the referral rate increased among patients aged ≥70 years.

### 3.4. Changes in Outcomes after the Prefecture-Wide CKD Initiative

**Figure 5:**
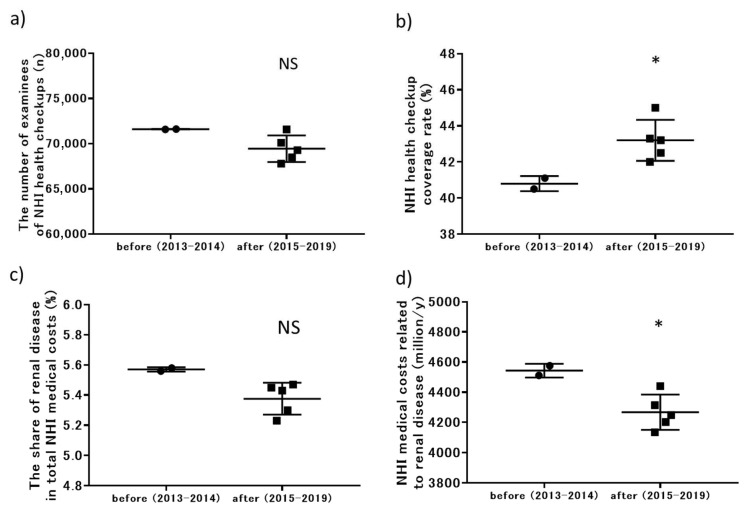
Changes in outcomes related to NHI before and after initiation of the prefecture-wide CKD initiative: (**a**) number of examinees who underwent NHI health checkups; (**b**) NHI health checkup coverage rates; (**c**) proportion of total NHI medical costs related to renal disease; (**d**) NHI medical costs related to renal disease. NHI, National Health Insurance; CKD, chronic kidney disease. * *p* < 0.05 versus before the initiative (2013–2014).

The factors related to NHI health checkups, including the number of examinees who underwent NHI health checkups, the NHI health checkup coverage rate, the proportion of total NHI medical costs related to renal disease, and NHI medical costs related to renal disease are shown in Table 1. The changes in these outcomes are shown in Figure 5. Compared with the period before the start of the CKD initiative (2013–2014), the NHI health checkup coverage rate increased significantly after the start of the initiative (2015–2019), from a mean (SD) of 40.8% (0.4) to 43.2% (1.1), with *p* = 0.04. After the start of the CKD initiative, the proportion of total NHI medical costs owing to renal disease tended to decrease, with marginal significance (*p* = 0.057). NHI medical costs related to renal disease decreased significantly after the start of the initiative, from a mean (SD) of 4543 (32) million JPY to 4268 (117) million JPY, with *p* = 0.03. Medical treatment rates for hypertension and diabetes in 2017 were 12.7% and 22.5%, respectively, and reached 13.3% and 24.3% in 2020.

**Figure 6:**
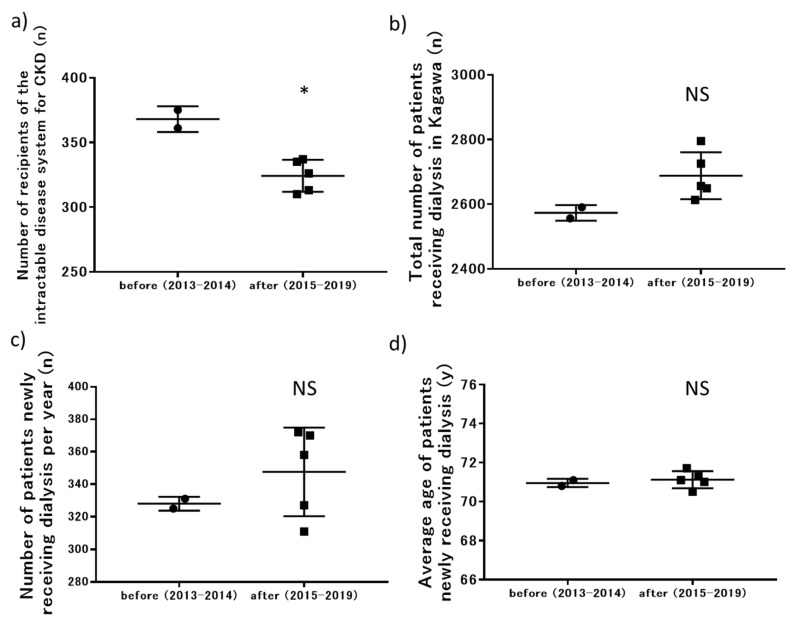
Changes in outcomes related to prefecture-wide data for all insurance-related factors after the start of the prefecture-wide CKD initiative: (**a**) number of recipients of the intractable disease system for CKD; (**b**) total number of patients receiving dialysis in Kagawa; (**c**) number of patients newly receiving dialysis per year; (**d**) average age of patients newly receiving dialysis. CKD, chronic kidney disease. * *p* < 0.05 versus before the initiative (2013–2014).

The following prefecture-wide data are shown in Table 2: the number of recipients of the intractable disease system for CKD, the total number of patients receiving dialysis, the number of patients newly receiving dialysis per year, and the average age of patients newly receiving dialysis. The changes in these factors are shown in Figure 6. The number of recipients of the intractable diseases system for CKD decreased after the start of the CKD initiative (*p* < 0.01). The total number of patients receiving dialysis increased gradually over the years (*p* = 0.09). The number of patients newly receiving dialysis per year varied widely from year to year, with no significant decrease during the first 5 years of the CKD initiative. There was no significant change in the average age of patients newly receiving dialysis before and after the start of the initiative.

## 4. Discussion

In this survey, we evaluated the prevalence of CKD among examinees who underwent NHI health checkups, the response to recommendations, and the medical costs in the first 5 years after the start of our prefecture-wide CKD initiative in Kagawa Prefecture. After initiation of the CKD initiative, we found an increase in the NHI health checkup coverage rate and participation by examinees in CKD-specific health guidance and medical visits and a decrease in NHI medical costs for renal disease. 

The overall prevalence of CKD increased over the years, but this increase was mainly observed in patients with early CKD, not in those with moderate-to-severe CKD. The number of patients with early CKD is expected to increase, especially among older adults. However, because the rate of progression to ESRD is low in older patients with early CKD [16], from the perspective of medical costs and patient health, CKD-specific lifestyle guidance alone may be sufficient for this group. As the CKD recognition and diagnosis increased, the treatment rates for diseases that are risk factors for CKD, such as hypertension and diabetes, also increased. We believe that this is a result of the special health checkup system.

The distribution of patients with CKD showed that most did not exhibit proteinuria, as previously reported [17]. As a limitation of this survey, eGFR values based on a single measurement of serum Cr are prone to misclassification, specifically in CKD G3a without proteinuria, thus not meeting the chronicity criterion. However, this single-measurement approach has often been used in CKD research [18,19,20]. For accurate assessment of proteinuria, quantitative assessment of proteinuria using the g/gCr method or albuminuria testing is preferable qualitative assessment using dipstick urinalysis. However, specific health checkups are inexpensive, simple, and permit broad detection of risk groups. In this initiative, we have established a system in which a wide range of risk groups are first detected using dipstick urinalysis health checkups, followed by a detailed quantitative proteinuria assessment by a general physician using the g/gCr method or albuminuria testing.

As renal function declines with age, the prevalence of CKD is thought to be higher in older people [21]. However, in this survey, the incidence of moderate-to-severe and severe CKD was higher in a relatively young population (40–69 years), which may be because CKD staging criteria differ by age. However, the prevalence of CKD among NHI recipients is high, compared with the prevalence in relatively young populations [22]; this may be because NHI recipients include people who are self-employed or do not have regular jobs, which may lead to unhealthy or irregular lifestyle habits. As we found that even relatively young people have a high risk, CKD initiatives are needed in the future, stratified by risk severity.

We previously reported the effects of an SGE program for preserving kidney function among examinees with early CKD [11]. A survey of the participation rate in group kidney disease management classes showed that the number of examinees who attended more than two consecutive classes was limited, with a participation rate of approximately 10%. In contrast, the NHI health checkup coverage rate increased after starting the CKD initiative. In addition to increasing the public’s interest in health, informing the public that CKD can be assessed in NHI health checkups may have contributed to the increased NHI health checkup coverage rate.

We also assessed the response of general physicians to the CKD initiative. The medical visit rate among examinees who receive a recommendation letter may reflect whether patients with moderate-to-severe CKD have a family physician. In some cases, even if an individual has a family physician, the attending physician may not return the recommendation letter signed. Therefore, the percentage of patients with CKD who have a family physician may be underestimated. Patients with moderate-to-severe CKD should have a family physician, even if they do not have comorbidities such as hypertension or diabetes. It is important to further increase the number of these patients who have a family physician. The referral rate to a nephrologist remained within the 10–20% range. This indicates that general physicians are not accurately following referral criteria, which may be the result of referring severe patients with employee insurance first or discouraging referral of older NHI patients.

In Japan, nephrotic syndrome, IgA nephropathy, polycystic kidney disease, and vasculitis syndrome are designated intractable diseases, but there is no medical cost support for kidney diseases caused by lifestyle-related factors such as diabetes and hypertension. The intractable disease designation for CKD in Kagawa Prefecture is the only one of its kind in Japan, and its existence may have an effect on CKD medical costs and clinical practice. The option to prescribe relatively expensive drugs for CKD, such as zirconium cyclosilicate, erythropoiesis-stimulating agents, hypoxia-inducible factor prolyl hydroxylase inhibitors, and sodium–glucose cotransporter 2 inhibitors at a fixed patient cost is expected to lead to adequate renal disease treatment and reduce the number of patients newly receiving dialysis.

We evaluated the prefecture-wide trend of patients who were newly undergoing dialysis, not limited to NHI-insured patients. In its 2018 report on renal disease initiatives, the MHLW aimed for a reduction in the annual number of new dialysis patients to 89% of the current level by 2028 [10]. In our survey, there was no apparent change in the number of patients newly receiving dialysis after the start of the CKD initiative. Formerly, Kagawa Prefecture had a relatively large number of nephrologists per capita and a relatively small number of patients undergoing dialysis per capita, which may be a reason for the difficulty in obtaining results with hard endpoints. 

In this study, we also analyzed the medical costs of NHI patients. NHI medical costs related to renal disease were defined using the ICD-10 codes 1401 (glomerular disease and tubulointerstitial disease) and 1402 (kidney failure). Therefore, NHI medical costs related to the renal disease include health care costs associated with dialysis as well as those associated with chronic glomerulonephritis and CKD. Although the results of a simple comparison between the two groups before and after the start of the CKD initiatives showed a downward trend in NHI medical costs related to renal disease, this downward trend may reflect bias, because the number of dialysis patients (a major cause of increased medical costs) did not decrease. Nevertheless, we believe that the downward trend in NHI medical costs related to renal disease in the intervention population is an important outcome. 

Several limitations of our survey should be noted. First, the total coverage of the NHI and the late-stage medical care system for older people is less than 50% of the total population of the prefecture, and the results do not reflect the prevalence of CKD among the general population. Second, we did not examine changes in kidney function and proteinuria as a result of recommendations for medical visits and lifestyle guidance for CKD. More detailed analysis is required in the future. Third, limitation of this program should be noted. The statistical methods used in this analysis had problems of short-term and before-after comparison. These problems need to be clarified in future long-term studies.

In conclusion, the present survey demonstrated that the prevalence of CKD has been increasing each year in Kagawa Prefecture; however, the NHI health checkup coverage rate has improved since the start of the prefecture-wide CKD initiative. We plan to continue this initiative to further reduce medical costs and the number of patients newly receiving dialysis in Kagawa.

## Figures and Tables

**Table 1:** Changes in factors related to NHI health checkups and medical costs.

	2013	2014	2015	2016	2017	2018	2019
Number of examinees undergoing NHI health checkups (n)	71,630	71,594	71,584	70,102	69,292	68,494	67,793
NHI health checkup coverage rate (%)	40.5	41.1	42.0	42.5	43.3	43.2	45.0
Proportion of total NHI medical costs for renal disease (%)	5.56	5.58	5.47	5.30	5.23	5.45	5.43
NHI medical costs related to renal disease (million JPY/year)	4511	4575	4440	4202	4134	4314	4248

NHI, National Health Insurance.

**Table 2:** Changes in the factors related to NHI health checkups and medical costs.

	2013	2014	2015	2016	2017	2018	2019
Recipients of CKD intractable disease system (n)	361	375	337	335	313	326	310
Total patients receiving dialysis in Kagawa (n)	2556	2590	2649	2613	2656	2725	2795
Patients newly receiving dialysis per year (n)	325	331	370	311	358	372	327
Average age of patients newly receiving dialysis (year)	70.8	71.1	71.0	70.5	71.3	71.7	71.1

NHI, National Health Insurance; CKD, chronic kidney disease.

## Data Availability

Not applicable.

## Supplementary Materials

The following are available online at https://www.mdpi.com/article/10.3390/jpm11111121/s1. Figure S1: Risk factor list chart for CKD used in the participatory structured group education program, in Japanese, Figure S2: Hanging banners to inform the public about CKD checkups provided as part of NHI health checkups, in Japanese, Figure S3: Prevalence of CKD in 17 cities and towns during 2017.
