# Supplementary material for: Changes in Prevalence and Health Checkup Coverage Rate of Chronic Kidney Disease (CKD) after Introduction of Prefecture-Wide CKD Initiative: Results of the Kagawa Association of CKD Initiatives
Source: J Pers Med. 2021 Oct 30;11(11):1121. doi: 10.3390/jpm11111121 (PMC8623378; doi:10.3390/jpm11111121)
Supplement: Supplementary file 1 [file jpm-11-01121-s001.zip › jpm-1428699-supplementary.pdf]

Supplementary Materials

Changes in Prevalence and Health Checkup Coverage Rate of Chronic Kidney Disease (CKD) after Introduction of Prefecture-Wide CKD Initiative: Results of the Kagawa Association of CKD Initiatives

Tadashi Sofue [1,*], Taiga Hara [2], Yoko Nishijima [3], Satoshi Nishioka [4], Hiroyuki Watatani [5], Masahito Yamanaka [6], Norihiro Takahashi [7], Akira Nishiyama [8], Tetsuo Minamino [1] and Kagawa Association of Chronic Kidney Disease Initiatives

1 Department of Cardiorenal and Cerebrovascular Medicine, Kagawa University, Takamatsu 761-0793, Kagawa, Japan; minamino.tetsuo.gk@kagawa-u.ac.jp
2 Department of Medicine for Community Healthcare Revitalization, Kagawa University, Takamatsu 761-0793, Kagawa, Japan; hara.taiga@kagawa-u.ac.jp
3 Department of CardioRenal Disease Regional Medicine, Kagawa University, Takamatsu 761-0793, Kagawa, Japan; nishijima.yoko@kagawa-u.ac.jp
4 Department of Nephrology, Takamatsu Red Cross Hospital, Takamatsu 760-0017, Kagawa, Japan; nsatoshi2400@yahoo.co.jp
5 Department of Nephrology and Rheumatology, Kagawa Prefectural Central Hospital, Takamatsu 760-8557, Kagawa, Japan; watatani@pa3.so-net.ne.jp
6 Department of Urology, Takamatsu Red Cross Hospital, Takamatsu 760-0017, Kagawa, Japan; ymasahito21@gmail.com
7 Department of Internal medicine, Shido-Akiyama Clinic, Takamatsu 769-2101, Kagawa, Japan; ntaka3658@yahoo.co.jp
8 Department of Pharmacology, Kagawa University, Takamatsu 761-0793, Kagawa, Japan; nishiyama.akira@kagawa-u.ac.jp
* Correspondence: sofue.tadashi@kagawa-u.ac.jp; Tel.: +81-(87)-891-2150; Fax: +81-(87)-891-2152
† Membership of the Kagawa Association of Chronic Kidney Disease Initiatives is provided in the Acknowledgments.

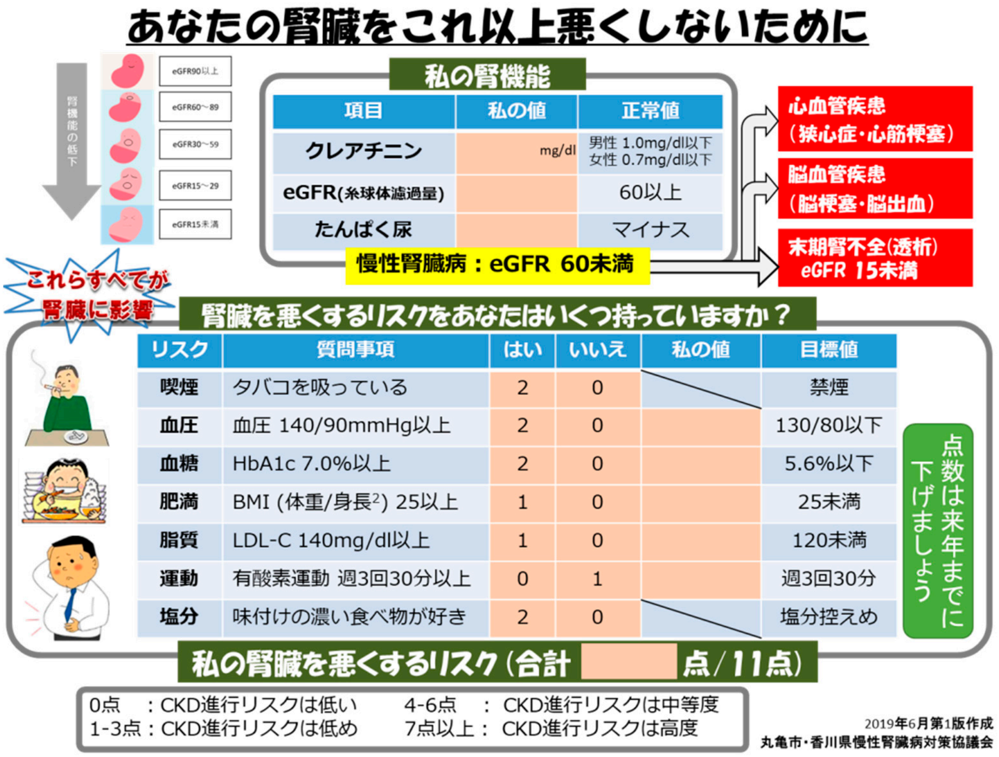

Figure S1. Risk factor list chart for CKD used by the participatory structured group education program (In Japanese).

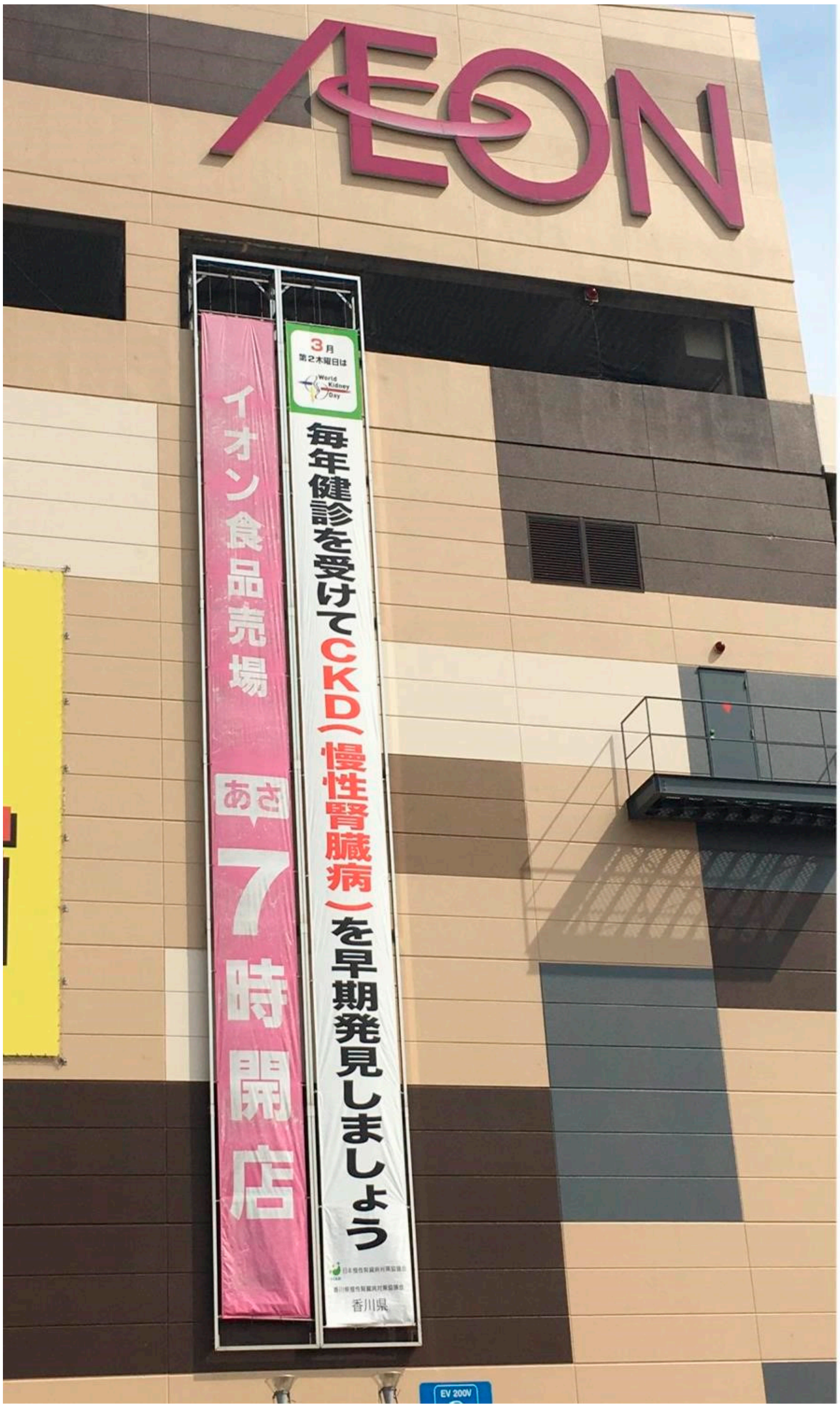

Figure S2. The hanging banners to inform the public that we provide CKD checkups as part of the NHI health checkups (In Japanese).

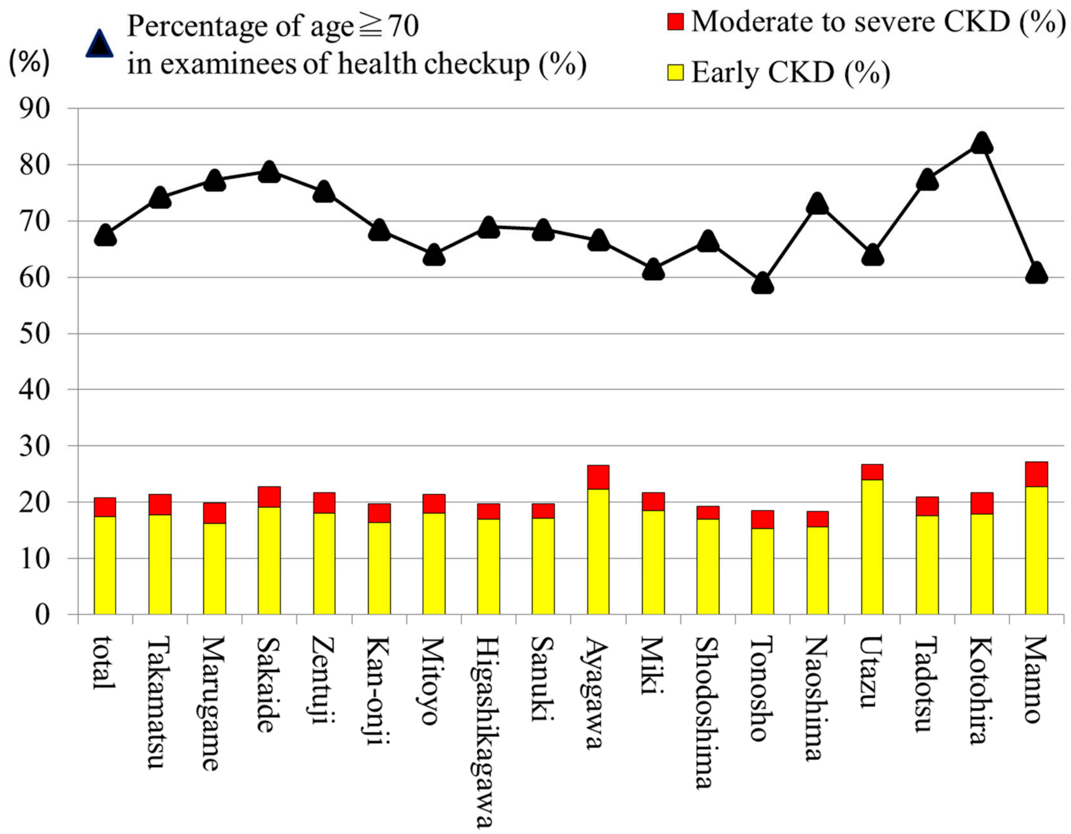

Figure S3. The prevalence of CKD among the 17 cities and towns in 2017.
